# Obstruction of Venous Drainage Linked to Transient Global Amnesia

**DOI:** 10.1371/journal.pone.0132893

**Published:** 2015-07-14

**Authors:** Ke Han, A-Ching Chao, Feng-Chi Chang, Chih-Ping Chung, Hung-Yi Hsu, Wen-Yung Sheng, Jiang Wu, Han-Hwa Hu

**Affiliations:** 1 Department of Neurology, First Hospital of Jilin University, Changchun, Jilin Province, China; 2 Department of Neurology, College of Medicine, Kaohsiung Medical University and Department of Neurology, Kaohsiung Medical University Hospital, Kaohsiung, Taiwan; 3 Department of Radiology, Taipei Veterans General Hospital and National Yang Ming University, Taipei, Taiwan; 4 Department of Neurology, Taipei Veterans General Hospital and National Yang-Ming University, Taipei, Taiwan; 5 Department of Neurology, Tungs’ Taichung Metro Harbor Hospital and Department of Neurology, School of Medicine, Chung Shan Medical University, Taichung, Taiwan; 6 Graduate Institute of Clinical Medicine and Department of Neurology, College of Medicine, Taipei Medical University and Hospital, Taipei, Taiwan; Charité University Medicine Berlin, GERMANY

## Abstract

Abnormal extracranial venous drainage modality has been considered an etiology of transient global amnesia (TGA). Evidence suggests that the transmission of the intrathoracic/intraabdominal pressure during a Valsalva maneuver (VM) is mainly through the vertebral venous system, and patency of internal jugular vein (IJV) is essential for venous drainage and pressure releasing. We hypothesize that obstruction of IJV venous drainage is a contributing factor in TGA pathogenesis. A magnetic resonance (MR) imaging protocol was used in 45 TGA patients and 45 age- and sex-matched controls to assess the morphologies of IJV, brachiocephalic vein (BCV) and asymmetry of transverse sinus (TS). The IJV was divided into the upper- and middle-IJV segments. Compared to the controls, TGA patients had significantly higher rates of moderate and severe compression/stenosis at the bilateral upper-IJV segment (left: 37.8% vs. 17.8%, P = 0.0393; right: 57.8% vs.15.6%, P<0.0012), in left BCV (60% vs. 8.9%, P<0.0004), and in TS hypoplasia (53.3%% vs. 31.1%, P = 0.0405). The prevalence of at least one site of venous compression/stenosis in IJV or BCV was significantly higher in patients than in controls (91.1% vs. 33.3%, P<0.0004). The diameter of the left TS in MRV, but not in T1 contrast imaging, was significantly smaller in TGA patients than in controls (0.31±0.21 vs. 0.41±0.19, P = 0.0290), which was compatible with downstream venous stenosis/obstruction. TGA patients have a higher prevalence of compression/stenosis of the bilateral IJV and the left BCV and TS hypoplasia, which is new evidence that supports the role of extracranial veins in TGA pathogenesis.

## Introduction

Transient global amnesia (TGA) is defined as a sudden and transient inability to acquire new information [1]. It can occur with certain triggering events or during Valsalva maneuver–like activities [1–3].

Cerebral venous congestion/hypertension has been considered a cause of TGA, which was attributed to venous reflux during a Valsalva maneuver (VM) consequent to valve incompetence of the internal jugular vein (IJVVI) [4–6]. Based on this theory, the deep cerebral veins draining the hippocampus are greatly suspected to be involved in TGA, and the hemodynamic changes of cerebral venous flow in these drainage veins can be shown at least during VM. Nevertheless, previous studies using ultrasound to study the intracranial venous hemodynamics, or using noncontrast venous magnetic resonance (MR) angiography to study the intracranial venous sinus morphology, failed to support this IJVVI theory [7–9].

On the other hand, internal jugular veins (IJVs) drain most of the brain venous outflow (BVO) when the patient is supine, whereas IJVs collapse and vertebral veins and intra-spinal veins drain most of the BVO when the patient is in the upright position [10–13]. Furthermore, evidences supported that the pressure from the chest and abdomen induced by VM was mainly transmitted to the epidural venous plexus, and, in turn, to the intracranium [13,14]. Thus, during the VM in sitting, the collapsed IJVs will re-open and drain BVO, which can be regarded as a protective mechanism to relieve intracranial venous congestion/hypertension [11,13]. Moreover, it had been observed that when the BVO is impaired by occlusion of the large venous trunks in the neck, a compensatory constriction develops in the major arteries of the brain [15], which may further impair the cerebral microcirculation. Therefore, we postulated that there are BVO obstructions [16–18] in TGA patients, which may weaken the protective mechanism to relieve intracranial venous pressure during VM and induce constriction of intracranial major artery. We used an MR-imaging protocol [18–22] in this study to investigate the BVO pathway obstruction in TGA patients.

## Methods

### Study design and participants

From January 2008 to December 2012, we prospectively recruited 45 patients with TGA from recurrent patients at the Neurology Department of the Taipei Veterans General Hospital. For this study, all of the patients were examined by a neurologist, and TGA was diagnosed according to the criteria, which were modified and validated by Hodge and Warlow [1]. For comparison, age- and sex-matched individuals were recruited prospectively as the control group from people receiving physical check-ups and who had no history of neurologic signs or symptoms. All TGA patients and control subjects underwent MR imaging studies and ultrasound study. Taipei Veterans General Hospital’s institutional review board approved the study protocol, and a written informed consent was obtained from all participants.

### MR imaging study

Contrast-enhanced magnetic resonance (MR) imaging using a 1.5T MRI (Excite II; GE Medical Systems, Milwaukee, WI, USA) were performed on TGA and age- and sex-matched controls, which included time-resolved imaging of contrast kinetics (TRICKS), contrast-enhanced axial T1-weighted MRI (Contrast T1), and phase-contrast based noncontrast enhanced magnetic resonance venography (MRV).

All patients and controls were examined within 10 days of their TGA attacks in a supine position and with the head in a neutral position. Contrast medium was injected with bolus through the superficial veins of the left-upper extremity to observe the cerebral venous return through transverse sinus (TS), sigmoid sinus, IJV-bulb, IJV, and brachiocephalic vein (BCV) to the superior vena cava by TRICKS. The injected dose of contrast media was 0.1 mmol/kg and the injected rate was 2 ml/sec with a power injector. The imaging sequences were axial T1-weighted and contrast-enhanced axial T1-weighted images extending from the skull to the thoracic inlet–level with imaging parameters: repetition time (TR) = 8.6 milliseconds, echo time (TE) = 2.5 milliseconds, inversion time (TI) = 400 milliseconds, flip angle = 15 degrees, slice thickness = 1.5 mm.

**Fig 1:**
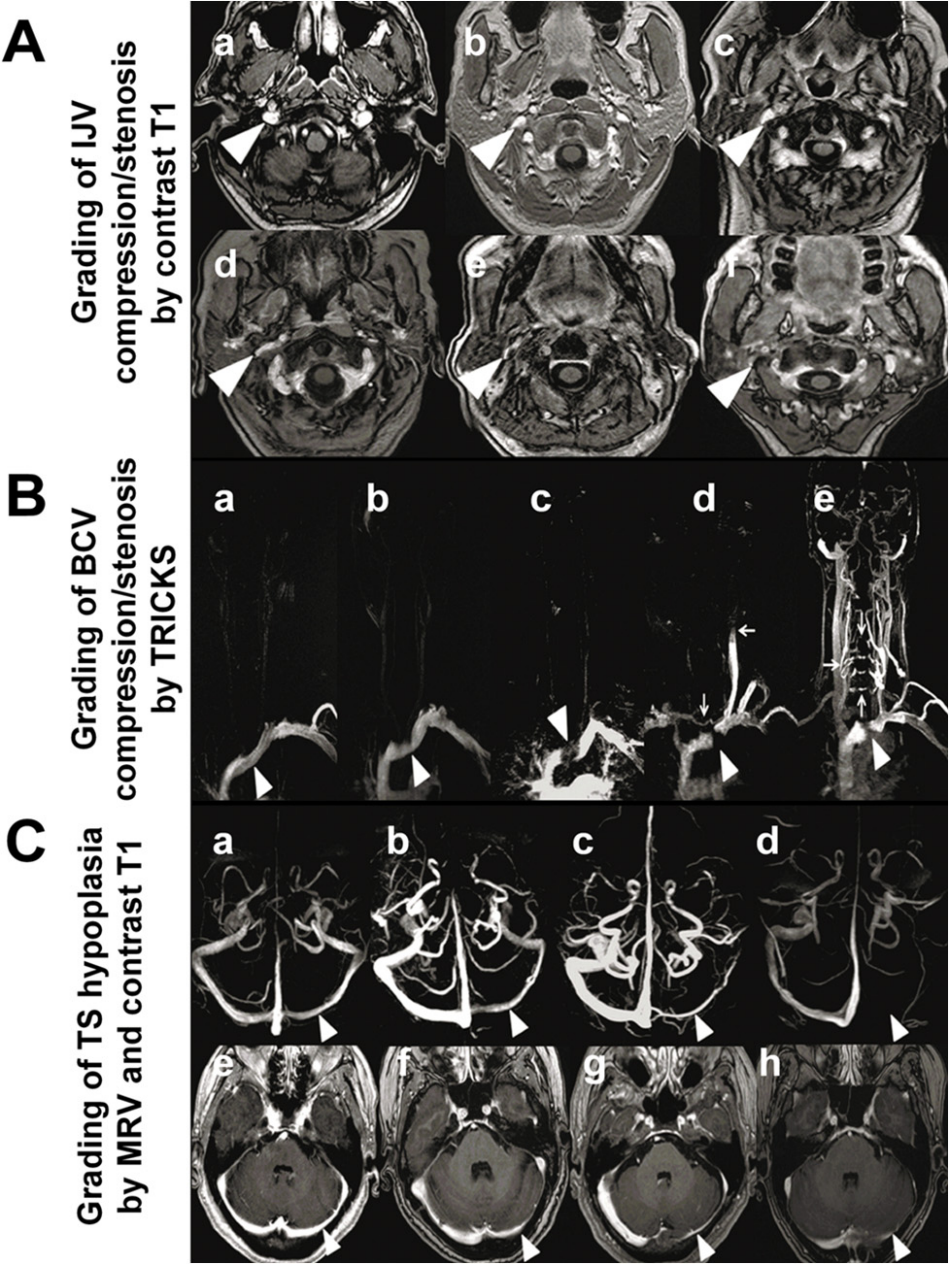
Grading of compression/stenosis using Contrast-enhanced *MR* (magnetic resonance) imaging. (A): Grading of *IJV* (internal jugular vein) compression/stenosis by *Contrast T1* (contrast-enhanced axial T1-weighted MR imaging). Grade 0: normal round or ovoid (a), Grade 1: mild flattening (b, c); Grade 2: moderate flattening (d); Grade 3: severe flattening (e), pinpoint (f) or not visualized. (B): Grading of left *BCV* (brachiocephalic vein) compression/stenosis by *TRICKS* (time-resolved imaging of contrast kinetics). Grade 0: normal (a, arrowhead), Grade 1: BCV with mild filling defect by the aortic compression (b, arrowhead); Grade 2: left BCV interrupted at the aortic arch (c, arrowhead) with filling defect, but without collateral; Grade 3: left BCV compression/occlusion (d-e, arrowhead) with different types of venous collaterals filling and reflux: Venous flow drains across the midline into the right IJV through the anterior cervical veins from left subclavian vein (d, vertical arrow); Reflux of IJV (d, horizontal arrow), the contrast medium injected from left subclavian vein appeared retrograde into left IJV; Collaterals of vertebral venous system, presence of collaterals of vertebral venous system, from the left subclavian vein draining directly through intrarachidian anastomoses to contralateral side at different levels (e, arrows). (C): Grading of *TS* (transverse sinus) asymmetry by *MRV* (magnetic resonance venography) (C a–C d). Grade 0: symmetrical TS (a); Grade 1: TS asymmetry≤50%(b); Grade 2: TS asymmetry >50% (c); and Grade 3 aplasia or signal absent (arrowhead pointing locations for comparison) (d). Grading of TS asymmetry by Contrast T1(C e–C h). Grade 0: symmetrical TS (e), Grade 1: TS asymmetry≤50% (f); Grade 2: TS asymmetry>50%(g); and Grade 3:aplasia or signal absent(arrowhead pointing locations for comparison) (h).

In this study, the IJV morphologies were assessed at the upper IJV (at C1–2 level) and the middle IJV (at C3–5 level) using Contrast T1. The IJV compression/stenosis was evaluated according to the following criteria by Zaharchuket al. [18]: grade 0 = normal round or ovoid appearance; grade 1 = mild flattening; grade 2 = moderate flattening; grade 3 = severe flattening or not visualized (Fig 1A).

Left BCV obstruction was graded according to the filling defect shown on TRICKS as follows: grade 0 = normal, or compression≤20%, grade 1 = compression >20% and≤80%, grade 2 = compression >80%, grade 3 = grade 2+ presence of different types of venous collaterals (Fig 1B) [19]. The vertebral/intraspinal/neck collaterals were evaluated by using TRICKS, focusing on the presence of posterior condylar veins and collaterals of vertebral venous system (Fig 1Bd and 1Be).

TS morphology was graded using MRV and Contrast T1, modified from the following criteria by Fofi et al. [22] grade 0 = TS symmetry or TS asymmetry with≤10% compared with the contralateral TS; grade 1 = TS asymmetry with>10% and≤50% compared with the contralateral TS; grade 2 = TS asymmetry with >50% compared with the contralateral TS; grade 3 = aplasia, or TS signal absent (Fig 1C). Hypoplasia was defined as the indexed TS being<50% of contralateral TS, and patients with such TS morphology were considered significant TS asymmetric or TS hypoplasia. The locations for measurement in MRV (Fig 1Ca–1Cd) and Contrast T1 (Fig 1Ce–1Ch) were illustrated.

All of the MR images were read by one neuroradiologist and one neurologist. Both were well trained in reading neuroimaging and were blinded to the subjects’ clinical characteristics. A consensus meeting was conducted to discuss any problems or disagreements. The intraclass correlation coefficient for grading was used to assess interrater agreement with an interrater reliability of 0.91.

### Statistical analysis

All values were expressed as mean ± standard deviation (SD) for continuous variables and number (percentage) for discrete variables. As the study design was matched case-control, the prevalence and severity of compression/stenosis of the bilateral IJV, BCV, and TS asymmetry were analyzed using conditional logistic regression to compare the differences between TGA patients and their matched controls. The Friedman test was used for continuous data. P value of <0.05 was considered significant. All analyses were performed with SAS 9.2.

## Results

The demographic data and clinical features of TGA patients and age-/gender-matched controls are summarized (Table 1). There were no significant differences in the vascular risk factors between TGA patients and controls. The comparisons of the grading of compression/obstruction/hypoplasia in various venous routes between TGA patients and controls with different modalities of MR imaging are summarized (Table 2).

**Table 1:** Demographic Data and Clinical Features of TGA Patients and Controls.

	TGA	Controls
	(n = 45)	(n = 45)
Age	61.5±8.7(35–85)	61.5±8.7(35–85)
Gender (M/F)	19/26	19/26
Coronary artery disease	1(2.2%)	0 (0%)
Hyperlipidemia	3(6.7%)	2 (4.4%)
Hypertension	4(8.9%)	5 (11.1%)
Diabetes Mellitus (DM)	2(4.4%)	1 (2.2%)
Headache with Cough	4(8.9%)	0 (0%)
Mitral ValveProlapse (MVP)	3(6.7%)	2 (4.4%)
Sleep Apnea Syndrome(SAS)	1(2.2%)	0 (0%)
Syncope	3(6.7%)	0 (0%)
Insomnia	1(2.7%)	0 (0%)
Glaucoma	2(4.4%)	0 (0%)
Carotid stenosis	0(0%)	0 (0%)
MCA stenosis	3(6.7%)	0 (0%)
Previous stroke	1(2.2%)	0 (0%)
Clinical profiles of TGA		
Recurrent		21(46.7%)
Duration of amnesia (hours)		7.5±7.9 (0.2–11)
VM–like activities or precipitating factor, n (%)		16(35.6%)

***TGA*** transient global amnesia, ***MCA*** middle cerebral artery, ***VM*** Valsalva maneuver, ***MCA stenosis*** included stenosis <50%.

**Table 2:** Comparison of the Degree of Venous Compression in the Internal Jugular Vein (IJV) and Brachiocephalic Vein (*BCV*) in Magnetic Resonance (*MR*) Imaging in TGA Patients and Controls.

	Side	Grading	TGA	Controls	P value
		group	(n = 45)	(n = 45)	
** **	** **	** **	TRICKS		
BCV Compression	Left	0 + 1	18 (40.9%)	41 (91.1%)	0.0004
** **	** **	2 + 3	27 (60.0%)	4(8.9%)	** **
			Contrast T1		
Upper IJV Compression	Left	0 + 1	28 (62.2%)	37 (82.2%)	0.0393
		2 + 3	17 (37.8%)	8 (17.8%)	
	Right	0 + 1	19 (42.2%)	38 (84.4%)	0.0012
		2 + 3	26 (57.8%)	7 (15.6%)	
Middle IJV Compression	Left	0 + 1	41 (91.1%)	41 (91.1%)	1
		2 + 3	4 (8.9%)	4 (8.9%)	
	Right	0 + 1	41 (91.1%)	40 (88.9%)	0.896
** **	** **	2 + 3	4 (8.9%)	5 (11.1%)	** **

***TGA*** transient global amnesia, ***TRICKS*** time-resolved imaging of contrast kinetics, ***BCV*** brachiocephalic vein, ***Contrast T1*** contrast-enhanced axial T1-weighted magnetic resonance imaging, ***IJV*** internal jugular vein, ***Upper IJV*** the IJV at C1–2 level, ***Middle IJV*** the IJV at C3–5 level.

### IJV compression/stenosis

Compression/stenosis of the IJV is common in the upper-IJV (focus on C1–2) and the middle-IJV (focus on C3–4) levels in Contrast T1. In comparison with the controls, there was significantly more moderate or severe IJV compression/stenosis in TGA patients at the bilateral upper-IJV level (left: 37.8% vs. 17.8%, P = 0.034; right: 57.8% vs.15.6%, P<0.0012), while there was no difference at the middle-IJV level. Furthermore, IJV with moderate or severe flattening or a non-visualization of venous caliber (grade2–3) in the TGA patients was more common on the right side (57.8%) than on the left side (37.8%) in the upper IJV, whereas there was no difference in the middle IJV (Table 2).

### Left BCV compression/stenosis

Severe compression with or without collateral formation of the left BCV by a TRICKS study was found in 27 (60%)TGA patients, which was significantly higher than that in controls (60% vs.8.9%, p = 0.0004). Of these 27 TGA patients, the presence of a vicarious shunt with posterior condylar collateral formation was found in 15 (55.6%) TGA patients in contrast to 8.9% in controls, while in 13 (48.1%) patients the contrast refluxed into the IJV (Fig 1Bd), or refluxed directly through intrarachidian anastomoses to the contralateral side at a different level (Fig 1Be).

**Fig 2:**
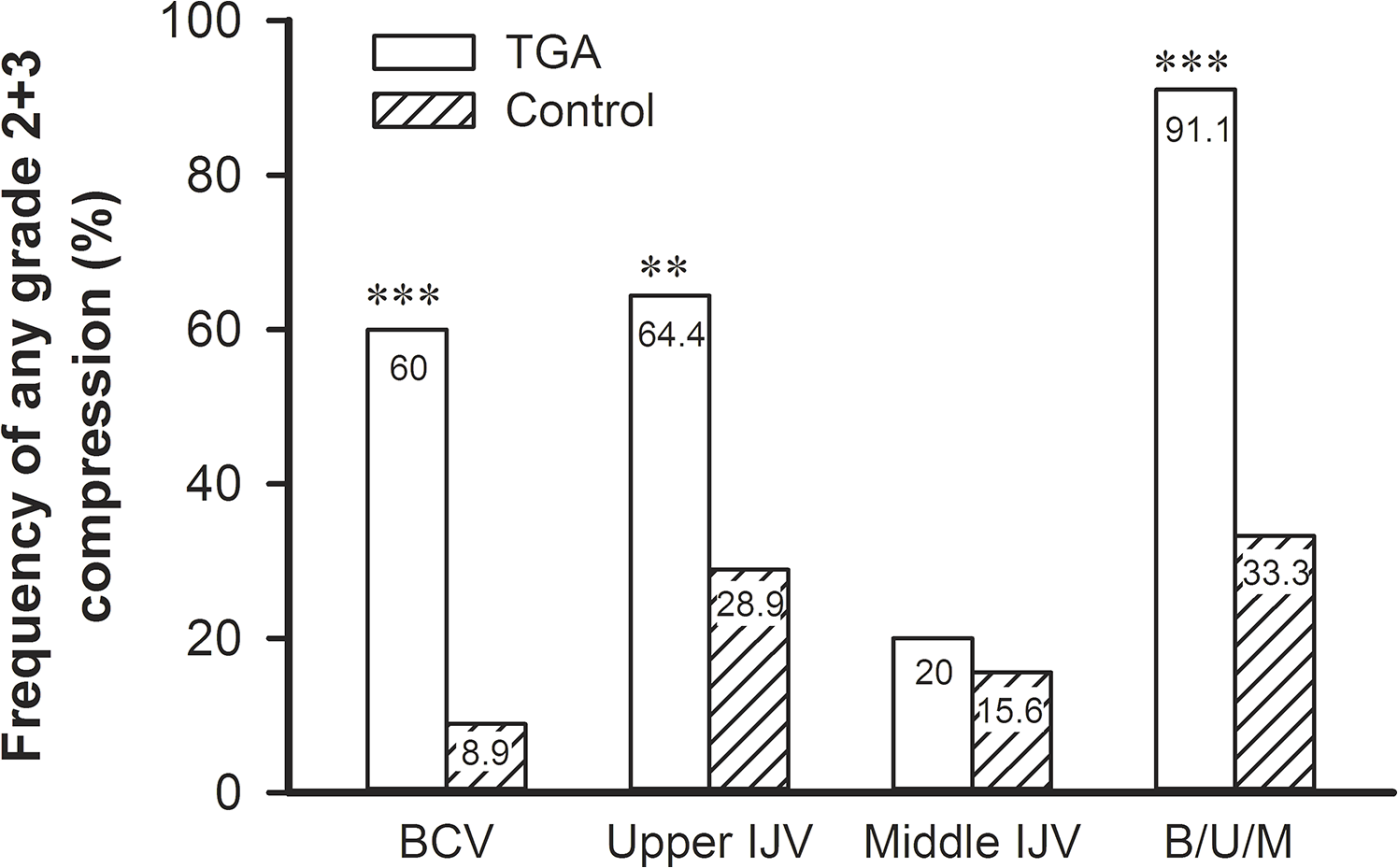
Differences between *TGA* (transient global amnesia) patients and controls in the frequencies of any significant venous compression of the venous outflow route on an individual basis. ** P*<0*.*01*, *** P<0.*001*. *BCV* left brachiocephalic vein compression, *IJV* internal jugular vein, *Upper IJV* Upper IJV compression at either side, *Middle IJV* Middle IJV compression at either side, *B/U/M*at least one site of venous compression/stenosis at left BCV or either side of Upper IJV or Middle IJV.

In summary, Fig 2 details the differences between TGA patients and controls in the frequencies of any significant compression/stenosis (grade 2+3) at outflow venous segments on an individual basis. Of note, the prevalence of at least one sign of venous outflow disturbance at IJVs and BCV was significantly higher in patients than in controls (91.1% vs.33.3%, P<0.0004).

### TS asymmetry or TS hypoplasia

The MRV study showed a significant difference in the TS asymmetry or TS hypoplasia between TGA patients and controls (62.2% vs. 31.1%, p = 0.0031) (Table 3). There was also a discrepancy in the measured TS diameters between MRV and Contrast T1 images, which demonstrated significantly smaller left TS in TGA patients than in controls (0.31±0.21 vs. 0.41±0.19, P = 0.0290) in MRV, but no such difference was revealed in T1 contrast imaging (Table 3).

**Table 3:** The Diameters of Transverse Sinus (TS) and TS h\Hypoplasia by Magnetic Resonance Venography (MRV) and Contrast-enhanced axial T1-weighted magnetic resonance imaging (Contrast T1) in TGA Patients and Controls.

	TGA		Controls	
	(n = 45)		(n = 45)	
	MRV	Contrast T1	MRV	Contrast T1
Left TS (cm)	0.31±0.21^&^	0.48±0.19	0.41±0.19	0.53±0.23
Right TS (cm)	0.60±0.18	0.72±0.19	0.58±0.13	0.68±0.15
TS Asymmetry (Left/Right)				
Grade 2+3	28 (62.2%)^#^	10(22.2%)	14 (31.1%)	10 (22.2%)
(TS hypoplasia)				

&, Left TS in MRV: TGA vs. Controls P = 0.0290.

#, TS hypoplasia in MRV: TGA vs. Controls P = 0.0031.

## Discussion

### Main findings

This study confirmed our hypothesis that TGA patients have a higher prevalence in greater severity of compression/stenosis of the bilateral IJV, left BCV, and TS hypoplasia by MR imaging, all of which may hinder the venous drainage from the cranium through IJVs to relieve the cerebral venous congestion/pressure [11,13] and cause cerebral vessel constriction [15] during VM while sitting. Our findings indicate that compression/obstruction of venous drainage is linked to the TGA, and they support the theory of TGA venous pathogenesis. To date our study is the first to describe the abnormal venous morphology from extrinsic compression at the IJV and BCV and TS hypoplasia in TGA patients.

### Cause and effect of IJV compression

We found that the upper-IJV compression was frequent among TGA patients, and the frequency was significantly higher than that in the controls. Nevertheless, for compression/stenosis at the middle IJV, the frequency in TGA patients was comparable to that in the controls. Although there have been reports about documented extrinsic structure impinging upon the vein at various levels of the internal jugular vein [18, 23–25] and causing neurological symptoms, such as headache and increase in intracranial pressure [25], such vein compression has never been reported and discussed in TGA patients in the literature, except for the one that reported brachiocephalic compression in TGA patients [19]. Jayaraman et al. reported that the styloid process or posterior belly of the digastric muscle, often adjacent to the lateral mass of C1, are the most common causes of extrinsic compression of the IJV [23]. Zaharchuket al. also found that IJV stenosis at the level of the C1 lateral masses and at the lower neck between the sternocleidomastoid and anterior scalene muscles were frequent findings in a cohort of MS patients [18]. The omohyoid muscle compression on IJV seems to be a possible cause of venous obstruction [24]. Our findings of the causes of extrinsic compression are similar to those of Jayaraman et al. and Zaharchuk et al.

**Fig 3:**
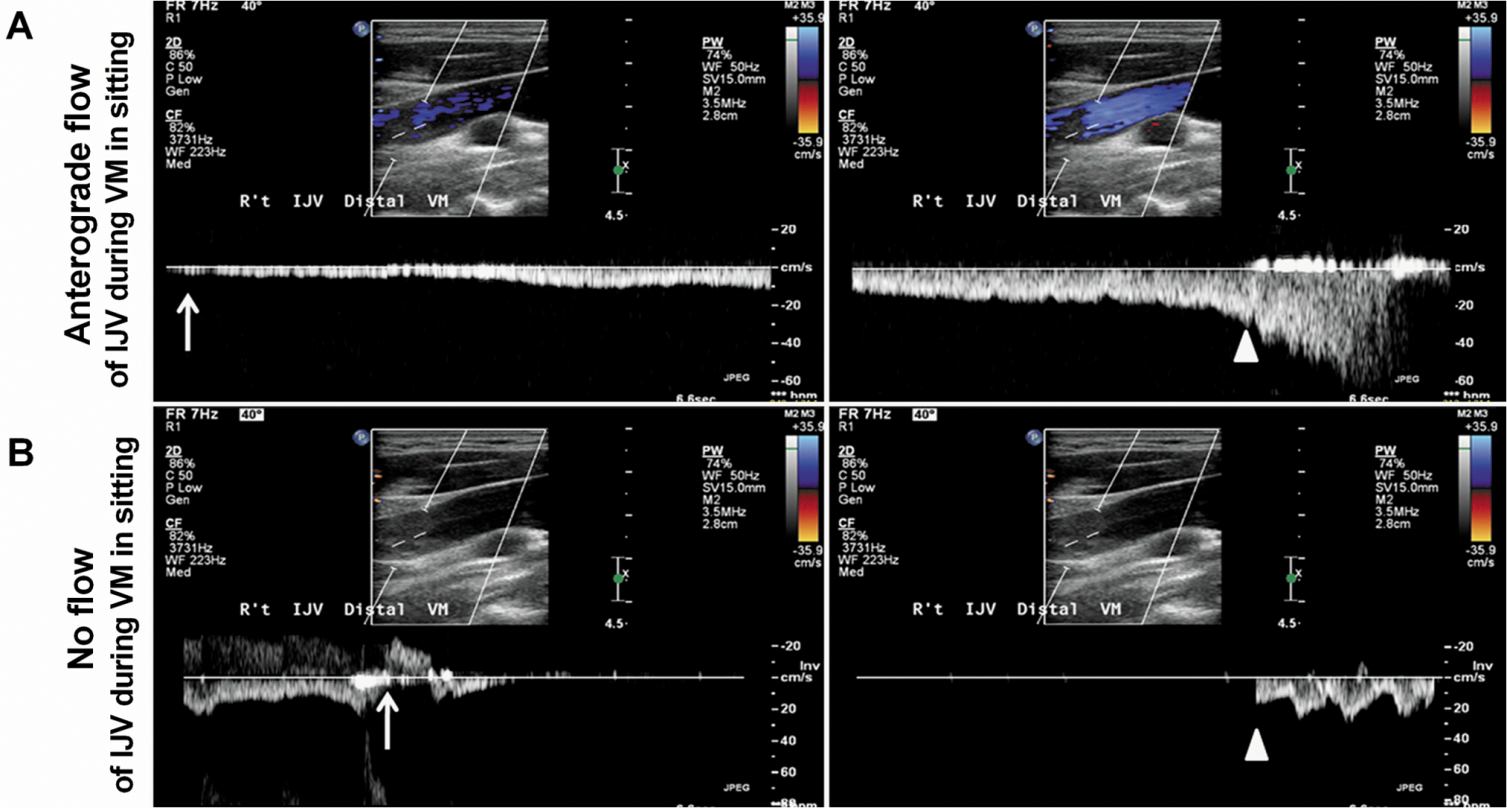
Display of Doppler spectra in *IJV* (internal jugular vein) during *VM* (Valsalva maneuver) in sitting. (A) Patient 1. Anterograde flow in right IJV during VM in sitting (upper panel, A) suggests the pressure can be released by the reopening IJV in a 42-year-old healthy woman with IJV patency. (B)Patient 2. No flow in right IJV during VM in sitting (lower panel, B)with transient venous reflux (arrow, B) indicates the intracranial venous pressure can’t be effectively relieved through the reopening of the IJV in a 53-year-old TGA patient with significant IJV stenosis/obstruction. Arrows indicate beginning of VM, arrowheads indicate end of VM.

The unfavorable hemodynamic effects of IJV compression/obstruction include longer circulation time of venous outflow or reduced blood flow through the IJVs compared to those without IJV stenosis [26–28]. In this study, we considered all the vein narrowing was due to extrinsic compression from the morphology of focal vein stenosis (Figs 1 and 3). In addition, these cases of stenosis were not considered as secondary to a recanalized thrombus because all of the patients in our study did not have any neurological symptoms before TGA attacks and after the resolving of TGA symptoms. In addition, all of these patients received a series of MR examinations, and no MR findings suggestive of a re-canalized thrombus were found. Moreover, we did the complete clinical work up for pre-thrombotic state TGA patients in our stroke team, because ischemic stroke in the posterior circulation should be considered and ruled out.

### Cause and effect of left BCV compression

The left BCV is vulnerable to extrinsic compression due to its long course across the midline anterior to the aortic arch into the superior vena cava. The left BCV is pinched and narrowed between the sternal notch and the major branches of the aortic arch, as first reported by Bok et al. in1978 [29].The compression is more significant at expiration or VM and relieved during deep inspiration [19,30–32]. The BCV compression with jugular reflux to the intracranium has been accidentally found in patients receiving dynamic brain scintigraphy or brain MR imaging [19,20,29,33], with the frequency of this phenomenon estimated at about 0·2% to 0·4% [19], and the association between BCV compression to TGA has been previously proposed by our team [19]. The current study confirms that there is a significantly higher frequency of significant BCV compression (60%) in our TGA patients as compared with 8·9% in the controls, which further indicates that the relationship between the occurrence of TGA attack and BCV compression might not be an accidental finding. We found that left BCV occlusion would cause retrograde venous flow through the left IJV to the intracranium or through the vertebral venous system to the contralateral side (Fig 1B). Both of the collateral venous routes are likely to enhance the intracranial pressure or venous congestion during VM.

### Cause and effect of TS hypoplasia

Asymmetry of TS is a common finding and is considered a normal variant, with hypoplasia or aplasia on one side in 20% to 39% of cases [22,34,35]. In this study, we arbitrarily define TS asymmetry≤50% (grade 0–1) to be physiological and not clinically significant and define TS asymmetry>50% (grade 2–3) as TS hypoplasia. In this study, we found that TS asymmetry >50% or TS hypoplasia was significantly more frequent in TGA patients than in controls from the MRV. Previous investigators reported hypoplasia or aplasia of TS in MRV to be associated with prolonged cerebral circulation time, impaired cerebral autoregulation, and linked to postcarotid-stenting hyperperfusion syndrome [36], severe brain edema in middle cerebral artery infarction [37], unruptured arteriovenous malformation [38] and associated to Parkinson’ in a cohort of Chinese patients [39]. TS hypoplasia has thus been considered having significant impact on cerebral hemodynamic regulation. For this reason, it is reasonable to suspect TS hypoplasia having a hemodynamic role in the TGA occurrence. Consistent with a previous study, there was a discrepancy in the measured diameter between MRV and Contrast T1 imaging [18]. If Contrast T1 imaging is considered the criterion-standard measurement, it is noteworthy that we found there was a significant difference in the diameter of left TS in MRV, i.e., significantly smaller left TS in TGA patients, and no such difference in Contrast T1 imaging (Table 3). An explanation to this may relate to the overestimation of narrowing of the venous route in the MRV study in the case of lower or slower flow. Thus, these findings are compatible with TGA patients having stenosis/obstruction of venous drainage from the cranium.

### A new evidence for venous theory in TGA and research perspective

The theory of intracranial venous hypertension as a consequence of IJVVI has been challenged [7–9], though we also found significantly higher frequency of IJVVI in TGA patients (data unpublished). The relationship between IJVVI and compression of IJV and BCV needs further evaluation.

We postulate that obstruction of venous drainage from the cranium is new evidence supporting the venous theory for TGA pathogenesis: in patients with IJV and/or BCV compression/obstruction, the vertebral venous system may serve as a significant venous route even when an individual is in a supine position, and it becomes the major route and drains most of the venous flow from cranium when sitting [10–14], while IJVs collapse. During the VM–like movement in sitting, the abundant venous flow in the vertebral venous system stops or reverses to the intracranium [11], draining up to the cavernous sinus through the basilar plexus and inferior petrous sinus [26,40]. Normally, the venous blood can be drained out via reopening and patent IJV to relieve the intracranial pressure and venous congestion. However, in TGA patients, an increase of intracranial pressure and venous congestion/hypertension in the basilar plexus and cavernous sinus ensue from lack of IJV patency. Moreover, venous stasis and occlusion may cause constriction of cerebral arteriole [15]. Although variation of the basal vein of Rosenthal, which drains the hippocampus, is common [41, 42], the basal vein of Rosenthal cannot go backward and upward to join the vein of Galen, so it drains directly into the cavernous sinus or basilar plexus. Thus, the VM–induced high venous pressure in the cavernous sinus or basilar plexus may affect the perfusion of either side of the hippocampus or bilateral hippocampus, resulting in venous ischemia to hippocampus or mesial temporal lobes and to TGA. The way of VM-induced pressure transmission to intra-cranium and variation of basal vein of Rosenthal may explain why the IJVVI theory was not supported by some previous studies [7–9].

TGA occurrence is generally not in a supine position, but it is difficult to perform an MR study with the patient sitting. In addition, we had tried VM and/or abdominal compression while performing TRICKS in 3 cases, but we found it was difficult to monitor the performance of patient’s doing VM, such as keeping intra-thoracic pressure around 40mmHg, maintaining head motionless and ensuring appropriate timing for simultaneous performance of TRICKS and VM. However, ultrasound studies have the advantage of studying the individuals in supine and sitting positions and during VM. In ultrasound examination, we can nicely demonstrate that reopening of the IJV during VM in a sitting position in a study subject (Fig 3A), and failure of such reopening of the IJV in another study subject due to IJV stenosis/obstruction (Fig 3B). Furthermore, the preliminary ultrasound data revealed that the flow volume of bilateral jugular veins in resting were significantly less in TGA patients when compared to controls, and the orthograde flow reappeared in the internal jugular vein during VM was also less often seen in TGA patients (unpublished data). These findings supported the hypothesis and findings of our current study. This MR imaging study could not clarify the role of IJVVI, such as to answer the question: could IJVVI also be a cause of failure of IJV reopening during VM? For these reasons, further studies with combined MR imaging and ultrasound are needed to evaluate the roles of IJVVI and venous obstruction. Finally, since the gold standard for any extracranial and intracranial vessels is a catheter angiogram, the stenosis of veins found in this study still need to be compare to catheter angiogram.

### Limitations

Our study does have limitations. 1. All patients and controls for MR imaging studies were in a supine position with the head in a neutral position. It is possible that patients’ turning their heads or patients in deep respiration might change the degree of venous route compression. 2. We did not correlate the ultrasound findings of IJV stenosis/compression with the MR findings because the IJV morphology examination with ultrasound has not been validated in our laboratory. 3. Of note, the recurrence rate of TGA was high in this study, and the reason may be due in part to racial differences, in that 60% of TGA patients in Taiwan have had obstruction of the left brachiocephalic vein (BCV), which we was rarely reported in any TGA studies or other studies with venous outflow impairment from Western society. Another reason may be due to the fact that many of our patients were referred from colleagues in Taiwan because of TGA recurrence. Therefore, interpretation of the results of this study should be done cautiously. However, other than the high recurrence rate, the clinical manifestations and MR imaging findings are similar between patients in our studies and patients in other studies.

## Conclusions

In summary, we confirmed that TGA patients have a high prevalence of unfavorable hemodynamic conditions, such as TS hypoplasia and compression/stenosis of the left BCV and the bilateral IJV, thereby indicating that venous drainage obstruction plays a significant role in the pathogenesis of TGA. There remains a need for further studies of the hemodynamic changes of cerebral arterial and venous blood flow, changes of the patterns of venous drainage during VM in a sitting position, and a comparison of those changes in patients with and without obstruction of the brain venous drainage route.

## Supporting Information

S1 Table Minimal Raw Data Set with Data Dictionary for TGA Study. (XLSX)
